# Supplementary material for: Regulation of reactive oxygen species during plant immunity through phosphorylation and ubiquitination of RBOHD
Source: Nat Commun. 2020 Apr 15;11:1838. doi: 10.1038/s41467-020-15601-5 (PMC7160206; doi:10.1038/s41467-020-15601-5)
Supplement: Supplementary file 1 — Supplementary Information [file 41467_2020_15601_MOESM1_ESM.pdf]

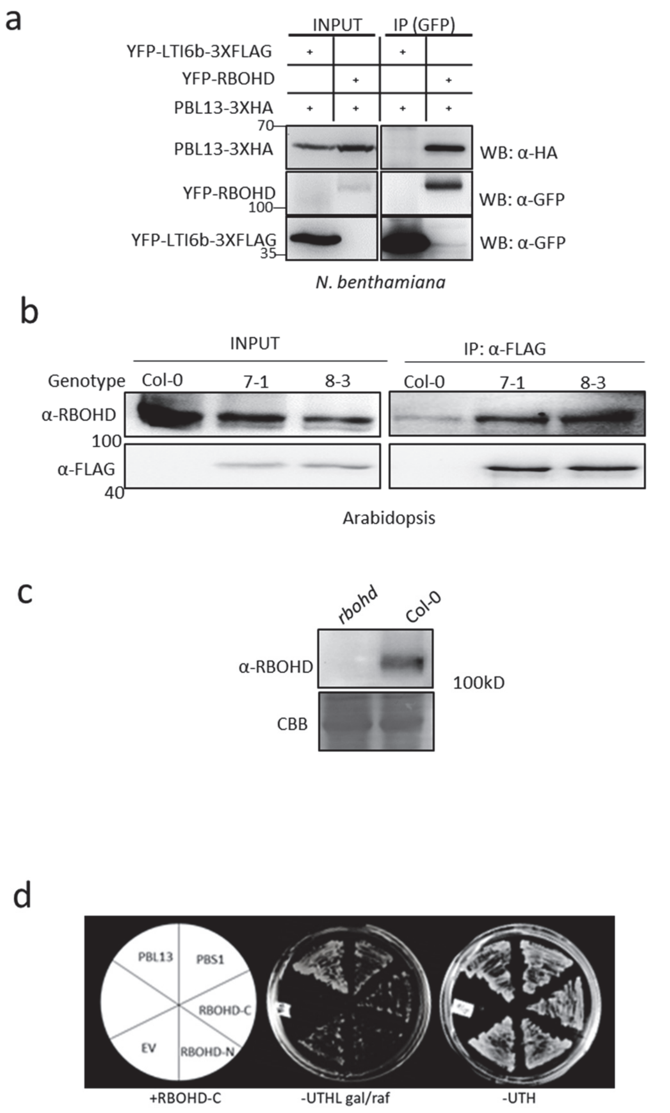

Supplementary Figure 1. PBL13 interacts with RBOHD. a. RBOHD associates with PBL13 in *N. benthamiana. PBL13-3xHA* was co-expressed with *YFP-RBOHD* or *YFP-LTI6b-3xFLAG* by Agrobacterium-mediated transient expression and subjected to co-immunoprecipitation using anti-GFP antibodies. PBL13 associates with RBOHD, but not the membrane-localized control LTI6B. **b.** RBOHD associates with PBL13 in Arabidopsis. Microsomal proteins were isolated from Col-0 or the *pbl13-2* knockout line complemented with wild type *PBL13-3XFLAG* (7-1 and 8-3) and subjected to co-immunoprecipitation using anti-FLAG antibodies. **c.** Detection of endogenous RBOHD *in vivo.* RBOHD antisera specifically recognizes RBOHD in Arabidopsis. The *rbohd* knockout line was used as a control. **d.** PBL13 interacts with RBOHD-C by yeast two-hybrid. RBOHD-C was cloned into pTBS1 (bait). PBL13, PBS1, RBOHD-N, RBOHD-C and the empty vector control were screened for interaction with pTBS1-RBOHD-C. Positive interactions were detected by streaking of yeast cells containing the indicated plasmids on SD medium lacking uracil, tryptophan, histidine, leucine (-UTHL) with galactose (gal) and raffinose (raf) as sugar sources.

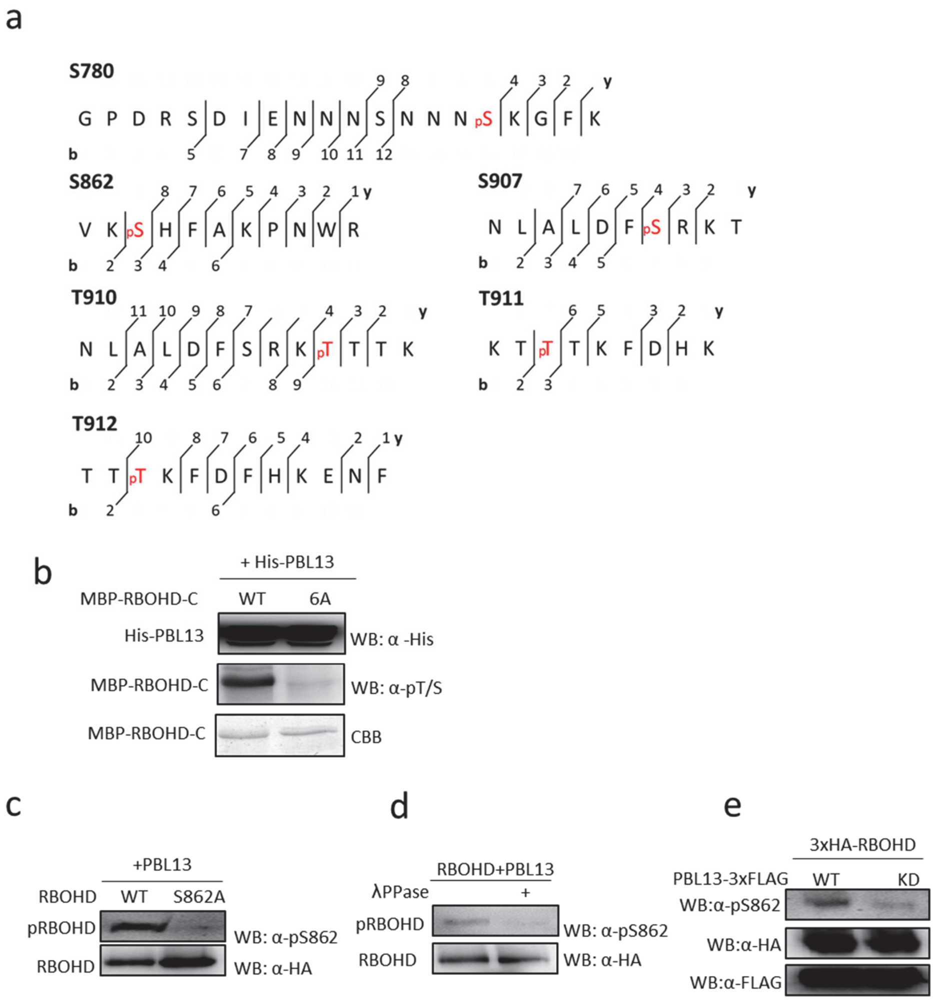

Supplementary Figure 2. PBL13 phosphorylates RBOHD's C-terminus. a. RBOHD-C phosphorylation sites. PBL13-mediated phosphorylation sites were identified from purified RBOHD-C after co-expression with PBL13 in *E. coli* by mass spectrometry. The vertical bars indicate the observed fragmentation sites of the precursor ion in the MS2 spectrum. **b.** The RBOHD-C phosphorylation null mutant 6A is not robustly phosphorylated by PBL13. His-PBL13 was co-expressed with MBP-RBOHD-C and MBP-RBOHD-C 6A (S78A, S862A, S907A, T910A, T911A, and T912A) in *E. coli* followed by purification of MBP-RBOHD-C. RBOHD phosphorylation was detected by immunoblot with α-pT/S. **c.** Phospho-antibodies for RBOHD pS862 specifically detect RBOHD WT, but not phosphorylation null RBOHD S862A after co-expression with PBL13 in *N. benthamiana*. 3xHA-RBOHD WT or S862A were immunoprecipitated with anti-HA and subjected to immunoblotting using anti-pS862. **d.** Phosphorylation of RBOHD is inhibited by lambda phosphatase (λPPase) treatment. Immunoprecipitated *3xHA-RBOHD* was treated with λPPase for 10min and subjected to immunoblotting using anti-pS862. The expression of RBOHD was detected by anti-HA immunoblotting. **e.** RBOHD phosphorylation is enhanced after co-expression with PBL13 (WT) compared to PBL13 kinase dead mutant (KD). 3xHA-RBOHD was co-expressed with *PBL13-3xFLAG* WT or KD and purified using anti-HA antibodies.

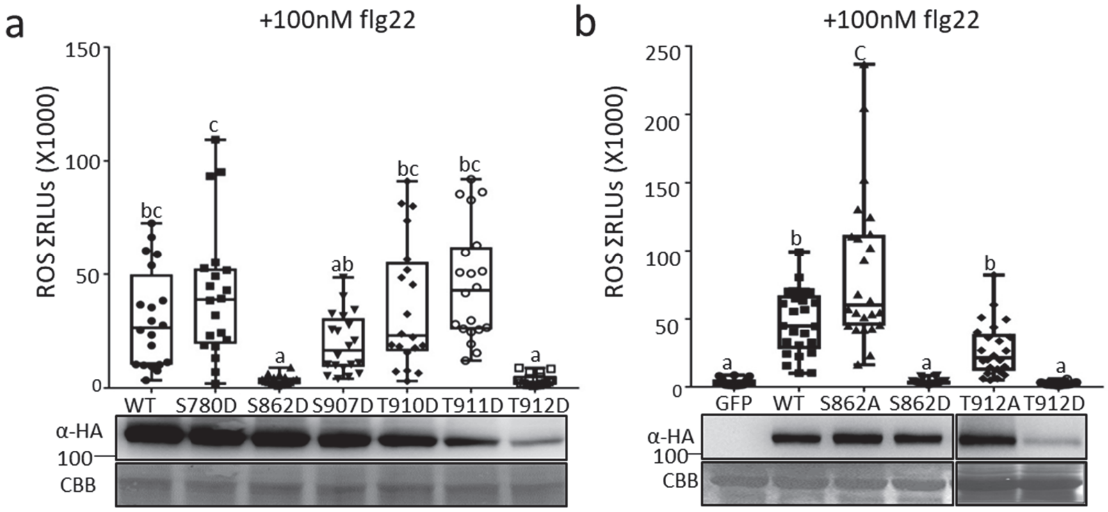

Supplementary Figure 3. Mimicking C-terminal phosphorylation of RBOHD affects stability and activity. a, b. *Nicotiana benthamiana NbRBOHB*-silenced plants were transiently complemented by expressing *GFP*, wild type, phosphonull or phosphomimetic mutants of *3xHA-RBOHD.* The complemented plants were treated with 100nM flg22 and the ROS burst was measured using a luminometer. For the box-plot, whiskers indicate minimum and maximum values, n>20, line indicates the median, the box boundaries indicates the upper (25th percentile) and lower (75th percentile) quartiles. Statistical differences were determined by ANOVA with post-hoc Tukey HSD test, α = 0.05, and letters indicate significant differences. The expression of RBOHD mutants was detected by anti-HA immunoblotting.

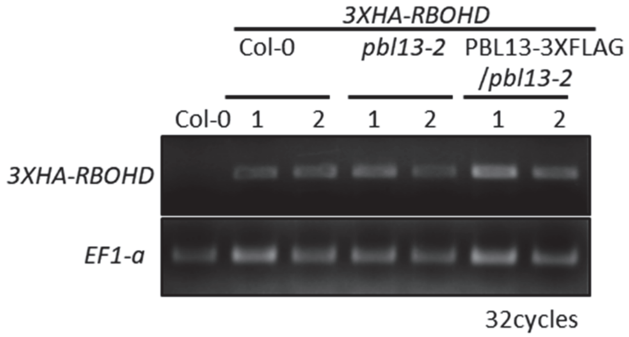

Supplementary Figure 4. *PBL13* does not affect the transcription of *RBOHD* in Arabidopsis. Total RNA was isolated from Arabidopsis transgenic lines *3xHA-RBOHD/Col-0 #3*, *3xHA-RBOHD/pbl13-2 #1, or 3xHA-RBOHD/PBL13-3XFLAG #3. 3xHA-RBOHD* transcripts were detected by RT-PCR with an HA specific forward primer and an RBOHD reverse primer. *ELONGATION FACTOR1-α* (*EF1-α*) was used as a control for gene expression. Numbers indicate independent biological replicates.

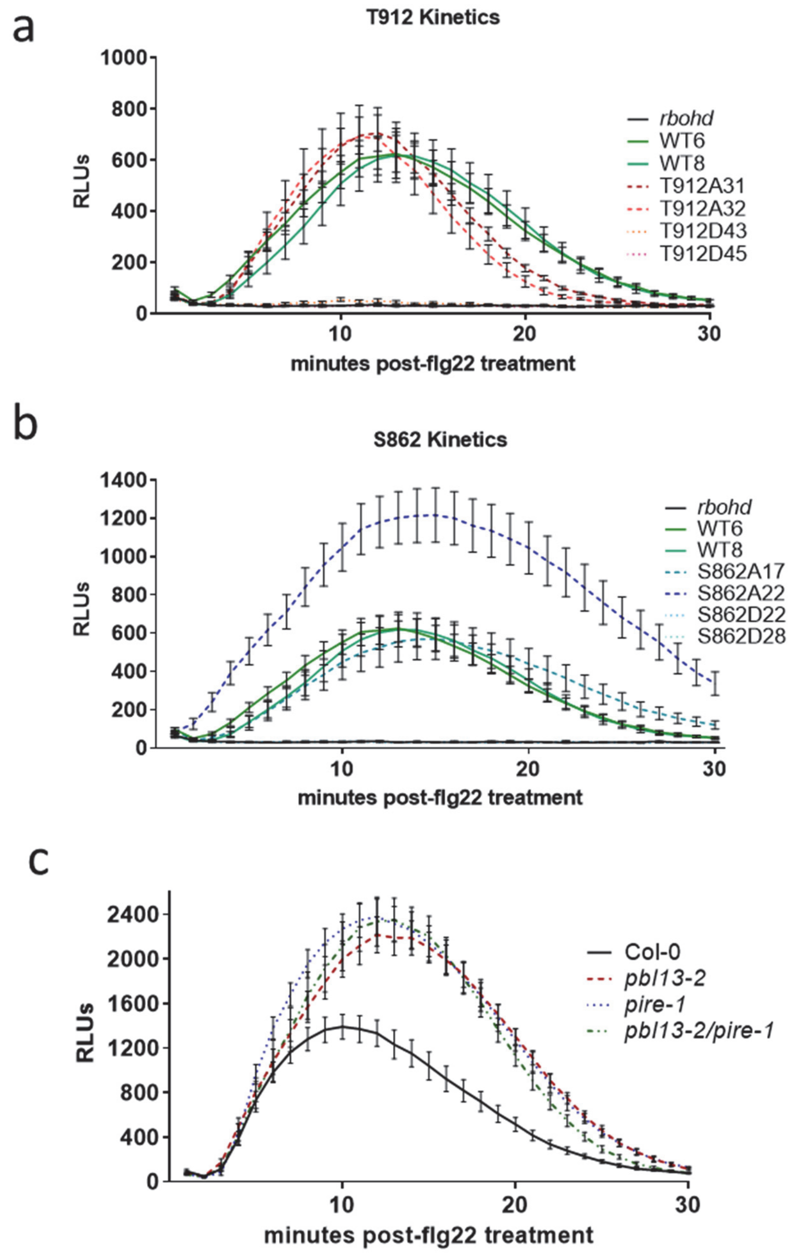

Supplementary Figure 5. Kinetics of the ROS burst in different genetic backgrounds. a. ROS burst after flg22 treatment in the Arabidopsis *rbohd* knockout, *rbohd* complemented with wild type (WT) *RBOHD,* as well as *rbohd* complemented with phosphonull $RBOHD^{T912A}$ and phosphomimic $RBOHD^{T912D}$. **b.** ROS burst after flg22 treatment in the Arabidopsis *rbohd* knockout, *rbohd* complemented with wild type (WT) *RBOHD,* as well as *rbohd* complemented with phosphonull $RBOHD^{S862A}$ and phosphomimic $RBOHD^{S862D}$. **c.** ROS burst after flg22 treatment in Arabidopsis Col-0, *pbl13-2, pire-1,* and *pbl13-2/pire-1* knockout lines. Plants were treated with 100nM flg22 and the ROS burst was measured using a luminometer. Results show the mean ±SE, n = 16 leaf disks.

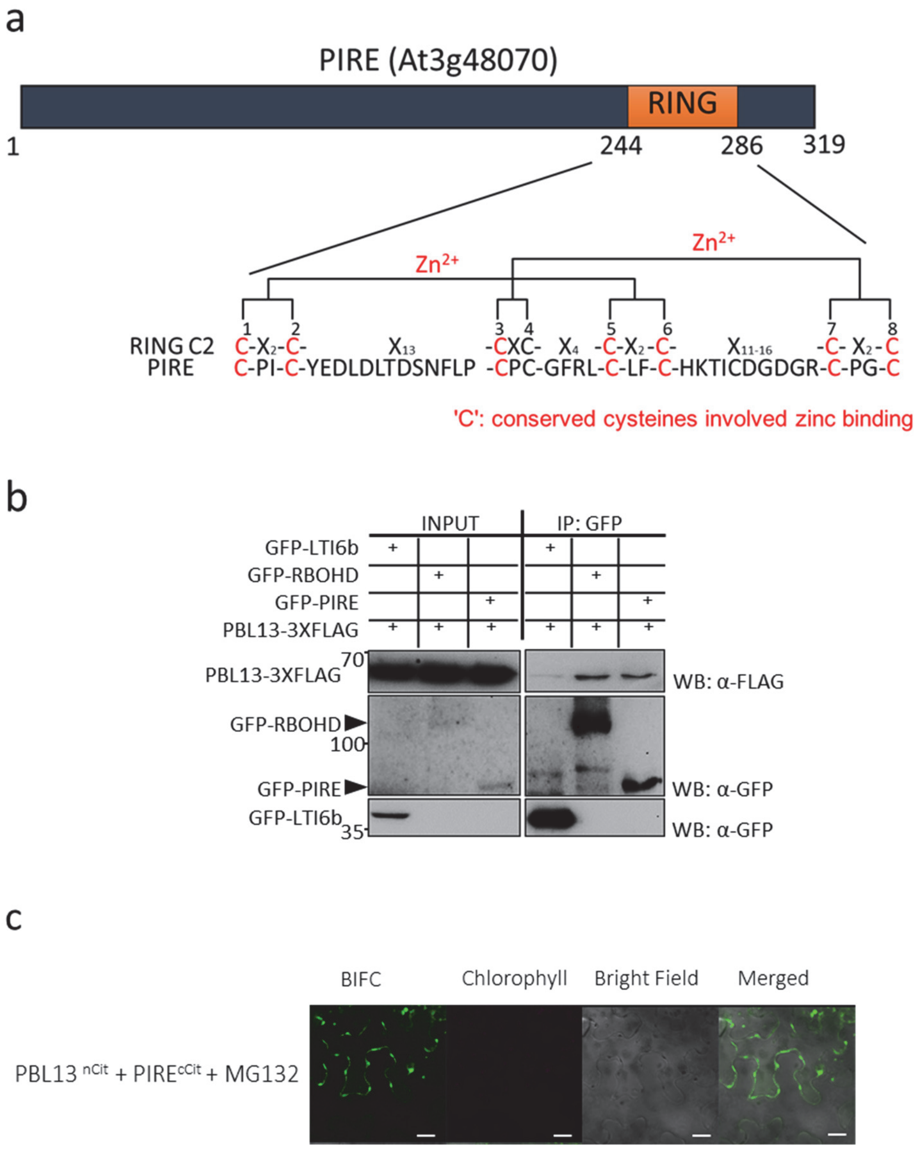

Supplementary Figure 6. PIRE is an RING E3 ubiquitin ligase that associates with PBL13. a. Domain architecture of PBL13-interacting RING E3 ubiquitin ligase (PIRE). The RING domain and conserved cysteine (C) residues involved in zinc binding are indicated. **b.** PBL13 associates with PIRE in Arabidopsis. PBL13-3xFLAG was co-expressed with GFP-PIRE, GFP-RBOHD or LTI6b-GFP in Arabidopsis Col-0 protoplasts and subjected to co-immunoprecipitation using anti-GFP followed by immunoblotting. PBL13 associates with PIRE and RBOHD, but not the membrane-localized control LTI6B. **c.** PIRE associates with PBL13 by BiFC. PIRE and PBL13 were transiently expressed in *N. benthamiana.* Scale bar=50μM.

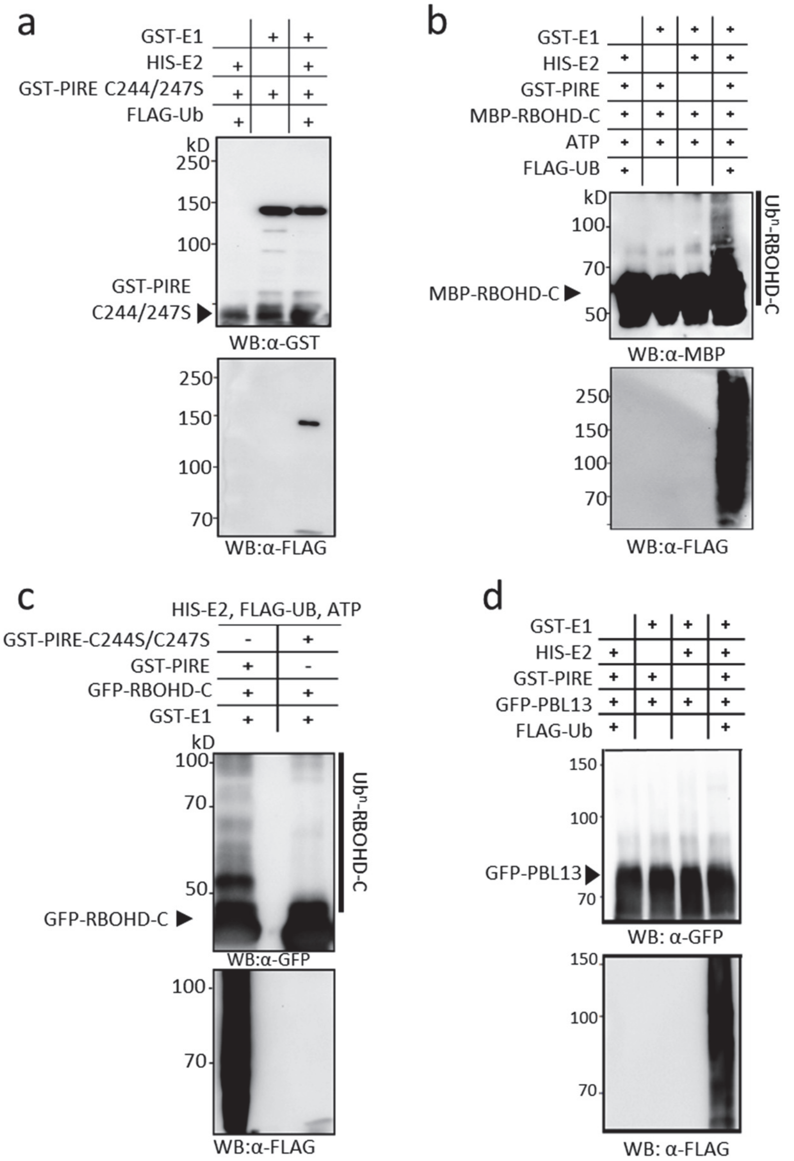

Supplementary Figure 7. PIRE RING domain activity is required for ubiquitination of RBOHD. a. The PIRE$^{C244S/C247S}$ mutant lacks E3 ligase activity. GST-PIRE$^{C244S/C247S}$ was incubated in ubiquitination assay buffer and no autoubiquitination was detected by GST immunoblot. The band on the FLAG immunoblot is ubiquitin transferred to GST-E1. **b.** Ubiquitination of MBP-RBOHD-C by PIRE. MBP-RBOHD-C was incubated with GST-PIRE in ubiquitination assay buffer containing E1, E2 and FLAG-tagged Ubiquitin (FLAG-Ub). Direct ubiquitination of MBP-RBOHD-C was detected by laddering after MBP immunoblotting. **c.** PIRE$^{C244S/C247S}$ is unable to ubiquitinate RBOHD-C. GFP-RBOHD-C was incubated with wild type PIRE or PIRE$^{C244S/C247S}$ in ubiquitination buffer. Direct ubiquitination of GFP-RBOHD-C was not detected by laddering after GFP immunoblotting. **d.** PIRE does not ubiquitinate PBL13. GFP-PBL13 was incubated with GST-PIRE in ubiquitination assay buffer. Higher molecular laddering was absent in GFP-PBL13 incubated with PIRE. Total ubiquitination detected by FLAG antibodies indicate active PIRE (bottom panel).

a

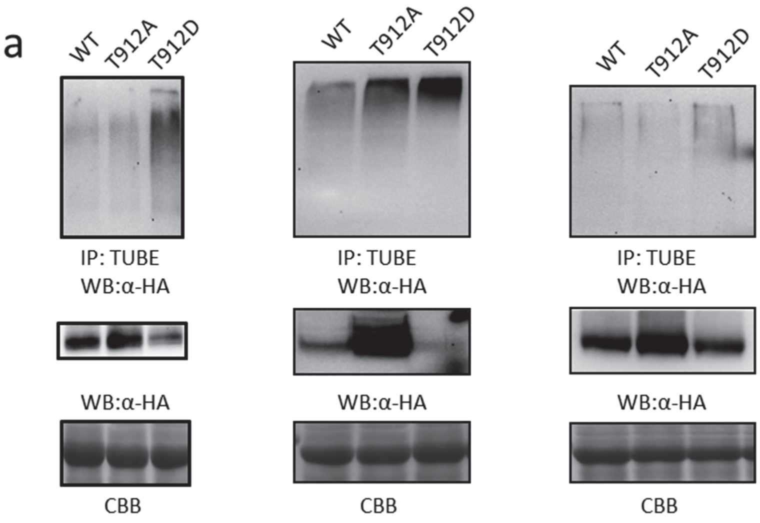

b

	rep1	rep2	rep 3	Average	ST DEV
WT	1	1	1	1	0
T912A	0.274879	0.423497	0.467061	0.388479	0.100763
T912D	7.215016	3.79748	2.693274	4.56859	2.357435

Supplementary Figure 8. TUBE immunoprecipitation. a. TUBE immunoprecipitation. RBOHD's ubiquitination was detected by TUBE assay after expressing 3xHA-RBOHD variants (WT, T912A, and T912D) in *Nicotiana benthamiana.* **b*.*** Ubiquitination was calculated by normalizing the levels of laddering with the amount of RBOHD present per sample in ImageLab.

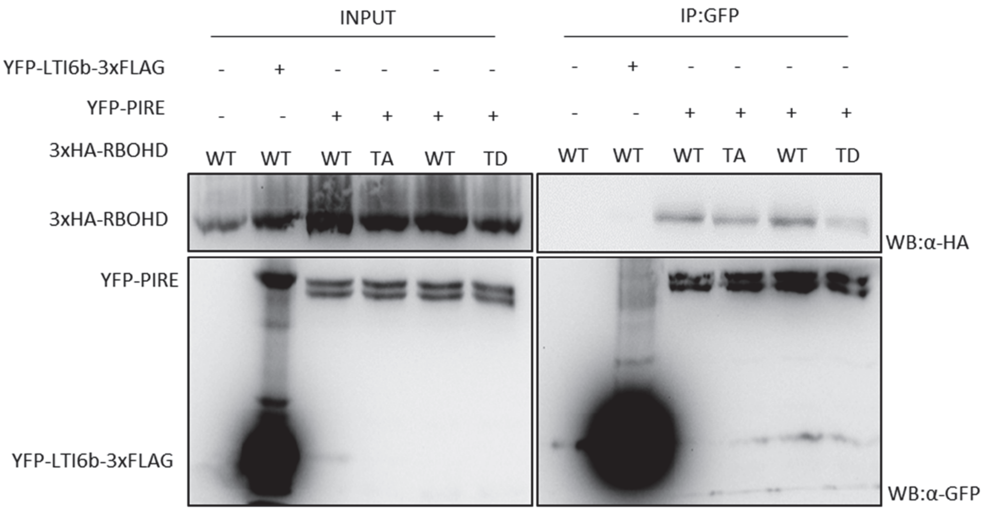

Supplementary Figure 9. RBOHD T912 phosphorylation mutants associate with PIRE. YFP-PIRE was co-expressed with 3xHA-RBOHD WT, T912A (TA), or T912D (TD) by Agrobacterium-mediated transient expression in *Nicotiana benthamiana*. Microsomal fractions were isolated and subjected to co-immunoprecipitation using anti-GFP.

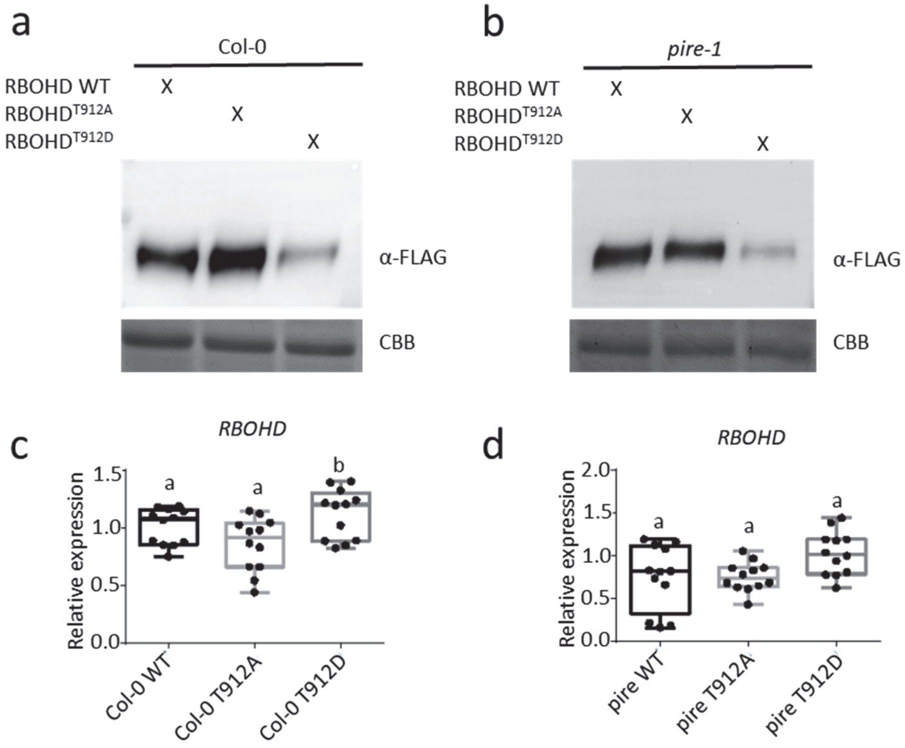

Supplementary Figure 10. Accumulation of RBOHD T912 mutants in Col-0 and the *pire-1* knockout. (a) Col-0 protoplasts were transfected with *FLAG-RBOHD, FLAG-RBOHD^{T912A}* and *FLAG-RBOHD^{T912D}*. Eighteen hours post-transfection, the accumulation of RBOHD variants was determined by anti-FLAG immunoblot (top panel). Coomassie brilliant blue (CBB, bottom panel). **(b)** *pire1-1* protoplasts were transfected with *FLAG-RBOHD, FLAG-RBOHD^{T912A}* and *FLAG-RBOHD^{T912D}*. RBOHD protein accumulation was determined as described above. (**c,d).** *FLAG-RBOHD* expression levels were measured utilizing RT-qPCR on transfected protoplasts as described in (a) and (b). Transfected Col-0 and *pire1* protoplasts were analyzed using the ΔΔcT method and normalized against Arabidopsis *ELONGATION FACTOR* 1α (AT2G18720). The primer for *FLAG-RBOHD* anneals to the C-terminal section of FLAG tag and N-terminus of *RBOHD*. Values represent relative expression compared to Col-0 WT or *pire1* WT, n = 12 from three biological replicates. For the box-plot, whiskers indicate minimum and maximum values, line indicates the median, the box boundaries indicates the upper (25th percentile) and lower (75th percentile) quartiles Statistical differences were determined by ANOVA with post-hoc Tukey HSD test, α = 0.05, and letters indicate significant differences.

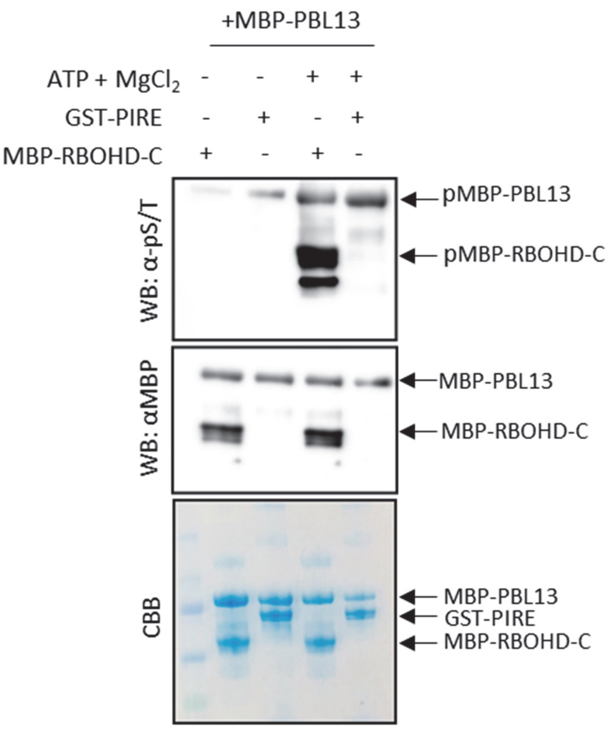

Supplementary Figure 11. PBL13 does not phosphorylate PIRE. *In vitro* phosphorylation was detected by incubating recombinant MBP-RBOHD-C or GST-PIRE with MBP-PBL13 followed by immunoblotting with anti-phospho S/T antibody. MBP-RBOHD-C's phosphorylation by MBP-PBL13 was detected in the presence of ATP.

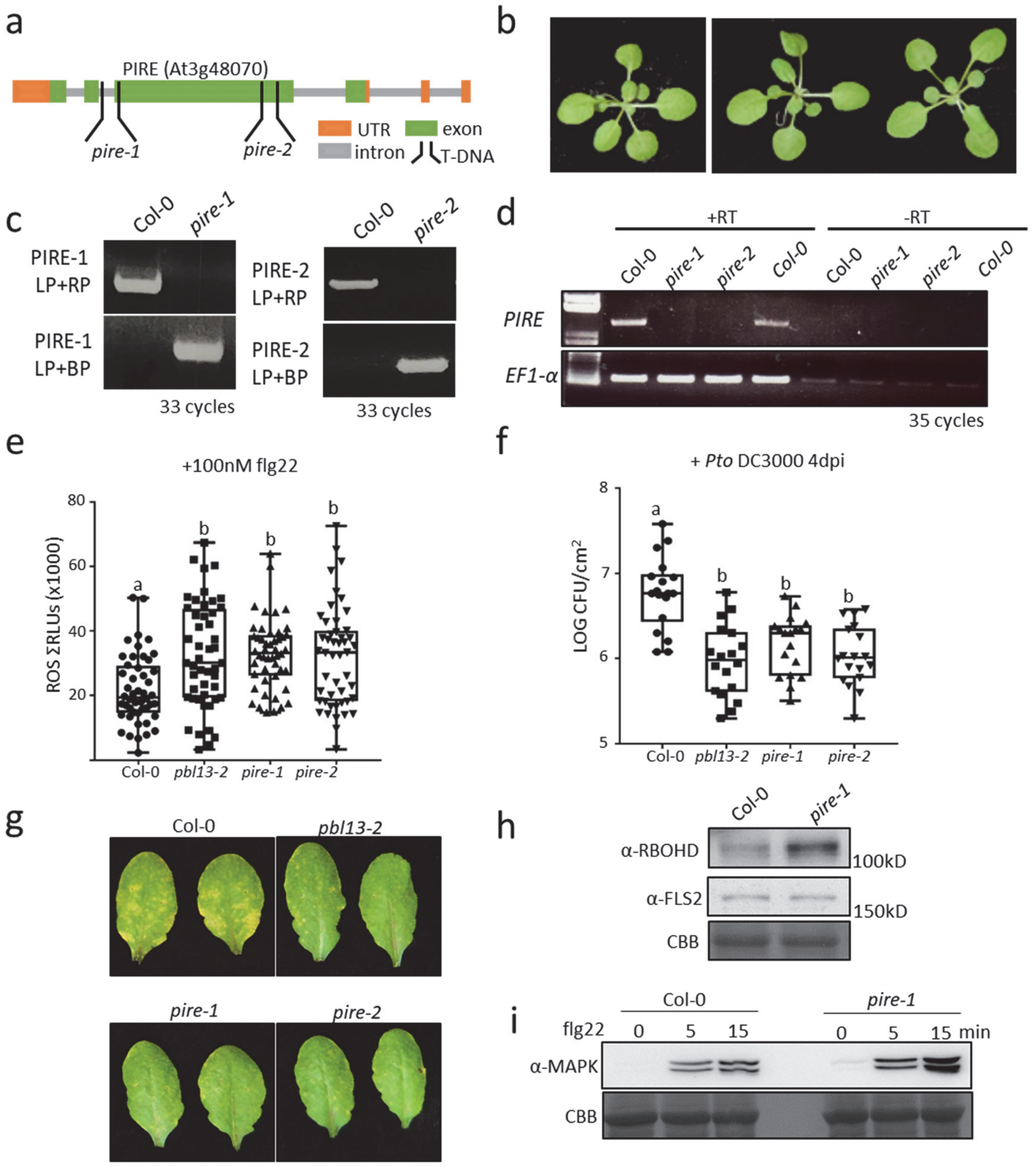

Supplementary Figure 12. Characterization of *pire* knockout (KO) lines. a. T-DNA insertion sites of *pire-1* (SALK_079510) and *pire-2* (SALK_138672), UTRs, exons, and introns are indicated. **b.** Growth phenotypes of *pire* knockouts. Image taken of three-week-old plants. **c.** Genotyping of the T-DNA insertion knock-out of *PIRE*. T-DNA insertion was detected by T-DNA specific primer (BP) and PIRE specific primers (LP). **d.** RT–PCR analysis of *PIRE*. Total RNA was isolated from Col-0, *pire-1*, or *pire-2.* The *PIRE* transcript was not detected in *pire-1* or *pire-2.* **e.** PIRE is a negative regulator of flg22 mediated ROS generation. ROS burst in Col-0, *pbl13-2*, and *PIRE* knockout lines. For the box-plot, whiskers indicate minimum and maximum values, n = 48, line indicates the median, the box boundaries indicates the upper (25th percentile) and lower (75th percentile) quartiles. Statistical differences were detected by ANOVA with post-hoc Tukey HSD test, α = 0.01, and letters indicate significant differences. **f.**

Quantification of *Pseudomonas syringae pv. tomato* (*Pto*) DC3000 titers four days post-infection (dpi). Bacterial titers were measured as log colony forming units (CFU) per cm^2 leaf tissue. Box-plots are as described in panel e, n=18. Statistical differences were detected by ANOVA with post-hoc Tukey HSD test, α = 0.01 and letters indicate significant differences. **g.** Disease symptoms after *Pto* DC3000 infection on indicated genotypes 4 dpi. **h.** *pire* knockout does not enhance FLS accumulation. RBOHD and FLS2 expression were detected by immunoblotting using RBOHD and FLS2 antibodies, respectively. **i.** *pire-1* exhibited enhanced MAPK activation after flg22 treatment. 7 day-old seedlings were incubated with 1uM flg22 as indicated duration. MAPK activation was detected by immunoblotting using p44/42 MAPK antibodies.

Supplementary Tables

Supplementary Table 1. Relative intensity of phosphorylated residues on RBOHD's C-terminus after co-expression with PBL13. Relative intensity of phosphorylated residues on RBOHD's C-terminus after co-expression with PBL13. MBP-RBOHD-C was co-expressed with His-PBL13 in *E. coli* and purified by MBP pulldown. The intensity of the phosphopeptide containing the indicated phosphosite was determined by the label-free MS1 quantification using peak area under the curve of each phosphopeptide in MaxQuant. The ratio of modification indicates the level of phosphorylation and higher value indicates more phosphorylation. The value was calculated by the ratio of phosphopeptide and unmodified peptide (ratio of phosphorylation= intensity of phosphopetide/ intensity of unmodified peptide). MBP-RBOHD-C's phosphorylation was not detected in the presence of empty vector (EV) and His-PBL13 kinase dead mutant (His-PBL13 KD).The location probability shown for each phosphosite is calculated by Andromeda algorithm integrated in MaxQuant.

Protein	Residue	Phosphorylation Probability on identified peptide	Ratio mod/base_rep1	Ratio mod/base_rep2
MBP-RBOHD-C	S780	GPDRDSDIENNNS(0.003)NNNS(0.997)KGFK	0.011633	0.00815
MBP-RBOHD-C	S862	VKS(1)HFAKPNWR	1.7981	1.638
MBP-RBOHD-C	S906	NLALDFS(1)RK	0.18032	0.21529
MBP-RBOHD-C	T910	KT(0.975)T(0.02)T(0.004)KFDFHK	0.044655	0.04305
MBP-RBOHD-C	T911	KT(0.022)T(0.881)T(0.097)KFDFHK	0.010749	0.052526
MBP-RBOHD-C	T912	T(0.041)T(0.041)T(0.917)KFDFHKENF	0.015884	0.015488

Supplementary Table 2. Primers used in this study

Primer Name	Sequence 5' to 3'	Purpose
PBL13-LP	CTACCTCAAGAAGTCGTCGTCCTC	Genotyping
PBL13-RP	GTGACCATGGATGTAATTTGCGGTTC	Genotyping
PIRE-LP	CACCATGATTTCTG ATTCGATCACC	Genotyping
PIRE-RP	TTCATGACCTGCAAAACATG	Genotyping
CACCRBOHD FW	CACCATGAAAATGAGACGAGGCAA	pENTR-RBOHD
RBOHD RV	CTAGAAGTTCTCTTTGTGGAAGTC	
RBOHD S780D FW	CAGTAACAACAATGATAAAGGGTTTAAGACAAGG	Site-directed mutagenesis
RBOHD S780D RV	CCTTGTCTTAAACCCTTTATCATTGTTGTTACTG	
RBOHD S862D FW	CGGGTACACGTGTCAAGGATCACTTCGCTAAACCTAAC	
RBOHD S862D RV	GTTAGGTTTAGCGAAGTGATCCTTGACACGTGTACCCG	
RBOHD S906D FW	CTAGCTTTGGATTTTGATCGAAAGACAACTACC	
RBOHD S906D RV	GGTAGTTGTCTTTCGATCAAAATCCAAAGCTAG	
RBOHD T910D FW	GCTTTGGATTTTTCTCGAAAGGATACTACCAAGTTTGACTTCCAC	
RBOHD T910D FW	GTGGAAGTCAAACTTGGTAGTATCCTTTCGAGAAAAATCCAAAG C	
RBOHD T911D FW	GGATTTTTCTCGAAAGACAGATACCAAGTTTGACTTCCAC	
RBOHD T911D RV	GTGGAAGTCAAACTTGGTATCTGTCTTTCGAGAAAAATCC	
RBOHD T912D FW	GATTTTTCTCGAAAGACAACTGATAAGTTTGACTTCCACAAAGAG	
RBOHD T912D RV	CTCTTTGTGGAAGTCAAACTTATCAGTTGTCTTTCGAGAAAAATC	
RBOHD S862A FW	CGGGTACACGTGTCAAGGCTCACTTCGCTAAACCTAAC	
RBOHD S862A RV	GTTAGGTTTAGCGAAGTGAGCCTTGACACGTGTACCCG	
RBOHD T912A FW	GATTTTTCTCGAAAGACAACTGCTAAGTTTGACTTCCACAAAGAG	

RBOHD T912A RV	CTCTTTGTGGAAGTCAAACTTAGCAGTTGTCTTTCGAGAAAAATC	
CACCRBOHD-C FW	CACCATGAAGGACATCATCAACAACATGAAA	pENTR-RBOHD-C
pGWB15 FW	GGGTTAATTAACATCTTTTACCC	RT-PCR
RBOHD250 RV	TGATGTCTAGCGTGATCTC	
CACCPIRE FW	CACCATGATTTCTGATTCGATCACCAACG	pENRT-PIRE and RT-PCR
PIRE RV	TCATGACCTGCAAAACATGCTAGACG	
EF1α FW	CTGGATTCGAGGGAGACAACATG	RT-PCR
EF1α RV	GCACCGTTCCAATACCACCAATC	
EF1α F	CTGGATTCGAGGGAGACAACA	qPCR
EF1α R	GCACCGTTCCAATACCACCAA	
RBOHD qPCR F1	CACGTGTCAAGTCCCACTTC	qPCR
RBOHD qPCR R1	TTGTCTTTCGAGAAAAATCCAAAGC	
FLAG_1For	GATTACAAGGACGATGACGATAAG	qPCR protoplasts
RbohD_Rev	TGGTGTCCGAGTTAGCTCCT	
CACCLTI6b FW	CACCATGAGTACAGCCACTTTCGTAG	pENRT-LTI6b
LTI6b RV	TCACTTGGTGATGATATAAAGAGCG	
CACCPBL13 FW	CACCATGGTTTTGTGTTTCCAAGATCCAG	pENTR-PBL13
PBL13 RV	GTACCGTTCCCCTCCGGCCTCGTT	
BamHI PBL13 FW	GGATCCATGGTTTTGTGTTTCCAAGATCC	pET28a-PBL13 and pET28a-PBL13ΔC
PBL13 XhoI RV	CTCGAGTCAGTACCGTTCCCCTCCG	
PBL13ΔC XhoI RV	CTAGCTCGAGTGAGGTAGGAACGGTGTAAGTGAA	
BamHI BIK1 FW	CAAGGATCCATGGGTTCTTGCTTCAGTTC	pET28a-BIK1
BIK1 SalI RV	CAAGTCGACCTACACAAGGTGCCTGC	
Mfe1 GW FW	GCACGAGATCTCACAAGTTTGTACAAAAAAGC	pMALC4x
GW HA HindIII RV	TTTCTTGTACAAAGTGGTGTACCCGTACGACGTTCCGGACTACG CTTAGAAGCTTACCTG	
His-MBP-BIK1_F	CTGTACTTCCAATCCAATATGGGTTCTTGCTTCAGT	MBP-BIK1
His-MBP-BIK1_R	CCGTTATCCACTTCCAATTTACACAAGGTGCCTGCCAAA	MBP-BIK1

His-MBP/GFP-PBL13_F	CTGTACTTCCAATCCAATATGGTTTTGTGTTTCCAA	MBP or GFP - PBL13
His-MBP/GFP-PBL13_R	TTATCCACTTCCAATTCAGTACCGTTCCCCTCCGGC	MBP or GFP - PBL13
GST_PIRE-F	CTGTACTTCCAATCCAATATGATTTCTGATTCGATCACC	GST-PIRE
GST_PIRE-R	CCGTTATCCACTTCCAATTTACAGATAAGCAAGGTTCTC	GST-PIRE
TBS1_PIRE_F	AAGGATGACGACGATAAGACCATGATTTCTGATTCGATCACC	Yeast two hybrid
TBS1_PIRE_R	TCACGCGGCCGCGCATGCCATTTACAGATAAGCAAGGTTCTC	Yeast two hybrid
TBS1-RbohD-Cterminus-F	ACAAGGATGACGACGATAAGACCATGAAGGACATCATCAACAAC	Yeast two hybrid
TBS1-RbohD-Cterminus-R	CTCACGCGGCCGCGCATGCCATCTAGAAGTTCTCTTTGTGGAAG	Yeast two hybrid
JG4-5_Bik1_F	TTATGATGTGCCAGATTATGCCTCTCCCGAAATGGGTTCTTGCTTCAGTTCTCGAGTCA	Yeast two hybrid
JG4-5_Bik1_R	CAAACCTCTGGCGAAGAAGTCCAAAGCTTCCTACACAAGGTGCCTGCCAAAAGGTTTTT	
JG4-5_RbohD_Cterminus_F	TGTGCCAGATTATGCCTCTCCCGAAATGAAGGACATCATCAACAAC	Yeast two hybrid
JG4-5_RbohD_Cterminus_R	TCCAAAGCTTCTCGAGTCGGCCGTCAGAAGTTCTCTTTGTGGAAGT	Yeast two hybrid
JG4-5_RbohD_N-terminus_F	TGTGCCAGATTATGCCTCTCCCGAAATGAAAATGAGACGAGGCAAT	Yeast two hybrid
JG4-5_RbohD_N-terminus_R	TCCAAAGCTTCTCGAGTCGGCCGTCATCTCTGCCAATTGTCAAGTA	Yeast two hybrid
JG4-5_PBS1-F	GTGCCAGATTATGCCTCTCCCGAAATGGGTTGTTTCTCGTGTTT	Yeast two hybrid
JG4-5_PBS1-R	CCAAAGCTTCTCGAGTCGGCCGCTACCCGGTACTGTTGCTCTCT	Yeast two hybrid
