# Supplementary material for: Regulation of reactive oxygen species during plant immunity through phosphorylation and ubiquitination of RBOHD
Source: Nat Commun. 2020 Apr 15;11:1838. doi: 10.1038/s41467-020-15601-5 (PMC7160206; doi:10.1038/s41467-020-15601-5)
Supplement: Supplementary file 3 — Reporting Summary [file 41467_2020_15601_MOESM3_ESM.pdf]

nature research

Corresponding author(s): Gitta Coaker

Last updated by author(s): Feb 17, 2020

Reporting Summary

Nature Research wishes to improve the reproducibility of the work that we publish. This form provides structure for consistency and transparency in reporting. For further information on Nature Research policies, see Authors & Referees and the Editorial Policy Checklist .

Statistics

For all statistical analyses, confirm that the following items are present in the figure legend, table legend, main text, or Methods section.

n/a	Confirmed	
☐	☒	The exact sample size (*n*) for each experimental group/condition, given as a discrete number and unit of measurement
☐	☒	A statement on whether measurements were taken from distinct samples or whether the same sample was measured repeatedly
☐	☒	The statistical test(s) used AND whether they are one- or two-sided<br>*Only common tests should be described solely by name; describe more complex techniques in the Methods section.*
☐	☒	A description of all covariates tested
☐	☒	A description of any assumptions or corrections, such as tests of normality and adjustment for multiple comparisons
☒	☐	A full description of the statistical parameters including central tendency (e.g. means) or other basic estimates (e.g. regression coefficient) AND variation (e.g. standard deviation) or associated estimates of uncertainty (e.g. confidence intervals)
☐	☒	For null hypothesis testing, the test statistic (e.g. *F*, *t*, *r*) with confidence intervals, effect sizes, degrees of freedom and *P* value noted<br>*Give P values as exact values whenever suitable.*
☒	☐	For Bayesian analysis, information on the choice of priors and Markov chain Monte Carlo settings
☒	☐	For hierarchical and complex designs, identification of the appropriate level for tests and full reporting of outcomes
☒	☐	Estimates of effect sizes (e.g. Cohen's *d*, Pearson's *r*), indicating how they were calculated

Our web collection on statistics for biologists contains articles on many of the points above.

Software and code

Policy information about availability of computer code

Data collection	no software was used
Data analysis	Data were analyzed in GraphPad/Prism

For manuscripts utilizing custom algorithms or software that are central to the research but not yet described in published literature, software must be made available to editors/reviewers. We strongly encourage code deposition in a community repository (e.g. GitHub). See the Nature Research guidelines for submitting code & software for further information.

Data

Policy information about availability of data

All manuscripts must include a data availability statement. This statement should provide the following information, where applicable:

- Accession codes, unique identifiers, or web links for publicly available datasets
- A list of figures that have associated raw data
- A description of any restrictions on data availability

The datasets generated during and/or analyzed during the current study are available from the corresponding author upon reasonable request.

Field-specific reporting

Please select the one below that is the best fit for your research. If you are not sure, read the appropriate sections before making your selection.

☒ Life sciences ☐ Behavioural & social sciences ☐ Ecological, evolutionary & environmental sciences

For a reference copy of the document with all sections, see nature.com/documents/nr-reporting-summary-flat.pdf

Life sciences study design

All studies must disclose on these points even when the disclosure is negative.

Sample size	Samples include at least four biological replications (plants) and repeated independently over time. Sample size was determined based on common sizes used in the literature and availability of growth space.
Data exclusions	No data were excluded from the analyses.
Replication	Experiments were replicated independently over time with each biological experiment replicated at least three separate times. Disease and ROS assays were repeated by different individuals in the laboratory to ensure reproducibility.
Randomization	Plants were grown in a completely randomized design for disease and ROS assays. For protoplast experiments, plant genotypes were grown individually in flats by genotype because protoplasts were combined by genotype prior to transfection.
Blinding	Investigators were blinded to the experimental genotype for ROS and bacterial growth assays. Investigators were not blinded for western blot analyses and co-IPs in order to ensure appropriate samples were run on particular lanes in the gel.

Reporting for specific materials, systems and methods

We require information from authors about some types of materials, experimental systems and methods used in many studies. Here, indicate whether each material, system or method listed is relevant to your study. If you are not sure if a list item applies to your research, read the appropriate section before selecting a response.

Materials & experimental systems

n/a	Involved in the study	
☐	☒	Antibodies
☒	☐	Eukaryotic cell lines
☒	☐	Palaeontology
☒	☐	Animals and other organisms
☒	☐	Human research participants
☒	☐	Clinical data

Methods

n/a	Involved in the study	
☒	☐	ChIP-seq
☒	☐	Flow cytometry
☒	☐	MRI-based neuroimaging

Antibodies

Antibodies used	Anti-RBOHD (Supplier: Agrisera, Sweden, AS15 2962)<br>Anti-HA-Peroxidase (Supplier: Sigma, 12013819001, clone 3F10)<br>Anti-GFP-Peroxidase (Supplier: Miltenyi Biotech, Germany, 130-091-833, clone GG4-2C2.12.10)<br>Anti-FLAG-Peroxidase (Supplier: Sigma, SAB4200119, clone 6F7)<br>Anti-MBP (Supplier: New England Biolabs, E8030S)<br>Anti-HIS (Supplier: Invitrogen, MA1-21315, clone AB_557403)<br>Goat-Anti-Rabbit IGg Peroxidase (Supplier: BioRad, 1706515)<br>Anti-mouse HRP (Supplier: BioRad, STAR77)<br>anti-pThreonine (Cell Signaling, 9381)
Validation	Anti-RBOHD was validated in the manuscript by demonstrating no cross-reactivity with other plant RBOHs using the rbohd knockout line. The antibody has also been validated on the manufacturer's website.<br>All other antibodies have been validated on the manufacturer's website and in multiple peer-reviewed publications. In addition, we have verified antibody specificity in plants by including negative controls, where no signal was detected in either Nicotiana or Arabidopsis in the absence of protein expression. These data indicate that the antibodies specifically detect epitope tagged proteins in plants.
